# Metastasis of Small Cell Lung Cancer to the External Auditory Canal: A Case Report

**DOI:** 10.1111/1759-7714.70059

**Published:** 2025-04-02

**Authors:** Toshiyuki Ito, Masamichi Yoshida, Hiroto Miki, Hiroki Goto, Shuuji Kodama, Atsushi Fujiwara, Hajime Fujimoto, Tetsu Kobayashi

**Affiliations:** ^1^ Department of Pulmonary and Critical Care Medicine Mie University Faculty and Graduate School of Medicine Tsu Mie Japan; ^2^ Department of Respiratory Medicine Mie Prefectural General Medical Center Yokkaichi Mie Japan

**Keywords:** external auditory canal, rare metastasis, small cell lung cancer

## Abstract

Patients with lung cancer often develop distant metastases; however, metastasis to the external auditory canal is rare. We report the case of a 77‐year‐old man who presented with left‐sided hearing loss, otalgia, and a red mass in the left external auditory canal. Computed tomography revealed masses in the left external auditory canal, lung, and pancreas. Histopathological analysis confirmed small cell lung cancer, with thyroid transcription factor 1 positivity in multiple lesions, suggesting a primary tumor in lung. Treatment with carboplatin and etoposide led to a reduction in metastatic lesions, including those in the external auditory canal, and improved hearing impairment. This case highlights a rare instance in which chemotherapy improved ear symptoms in a patient with small cell lung cancer metastasis to the external auditory. Written informed consent was obtained from the patient for the publication of this case report and accompanying images.

## Graphical abstract

We report a rare case in which chemotherapy improved ear symptoms in a patient with small cell lung cancer metastasis to the external auditory canal. Multimodal therapy is often required for the treatment of cancer to the external auditory canal, whereas chemotherapy alone for external auditory canal metastasis with high sensitivity to anticancer drugs is suggested to improve ear symptoms and quality of life.

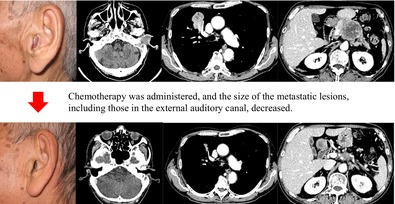

## 1 Introduction

Patients with lung cancer frequently develop distant metastases; however, metastasis to the external auditory canal (EAC) is rare. Although the estimated annual incidence of 2.2 million cases, only three case reports have described metastasis to the EAC, and its exact frequency remains unclear [1, 2, 3, 4]. Moreover, the clinical course of lung cancer metastasis to the EAC remains poorly understood. This report describes a rare case in which chemotherapy was effective, leading to the improvement of ear symptoms associated with lung cancer metastasis to the EAC.

## 2 Case Report

**FIGURE 1:**
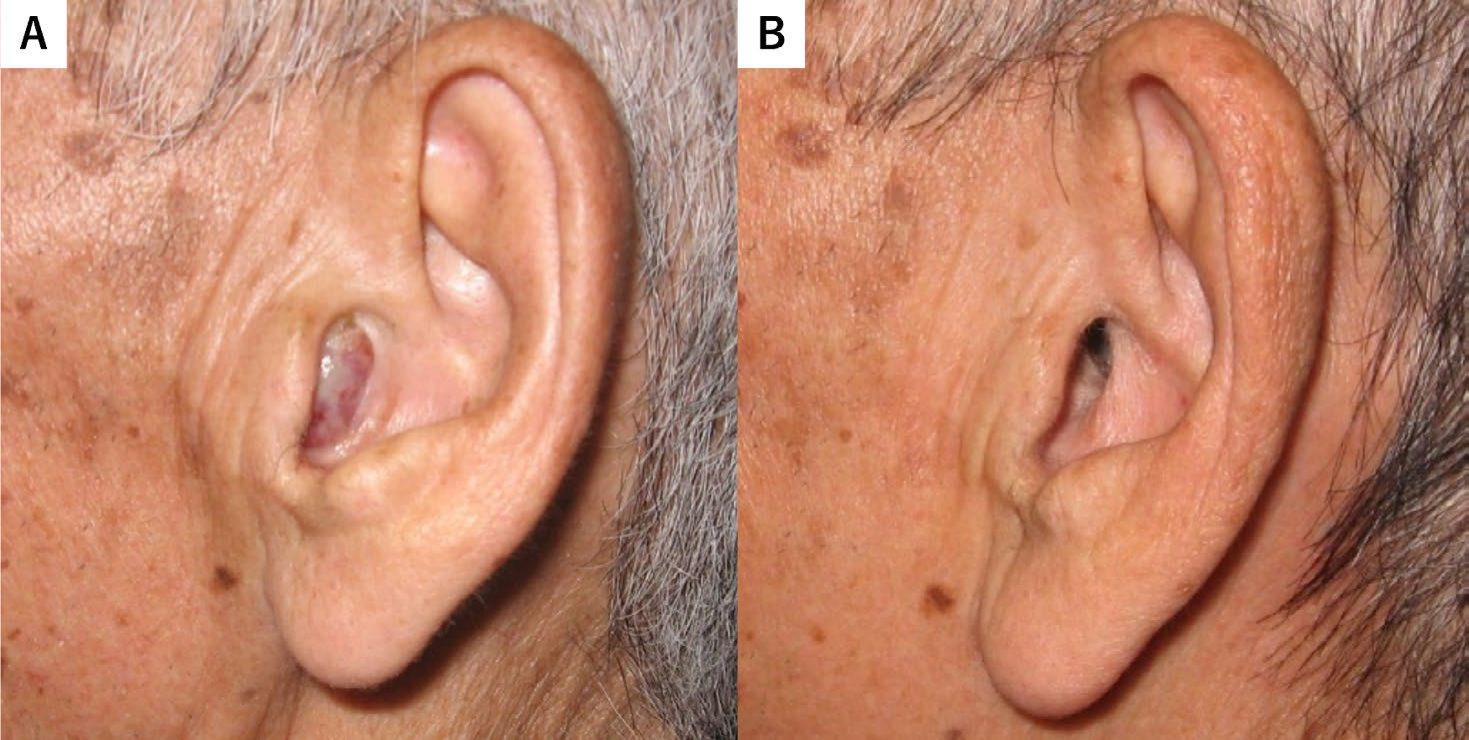
Left external ear findings. (A) Red mass occupying the external auditory canal before chemotherapy. (B) Regression of the mass after chemotherapy.

**FIGURE 2:**
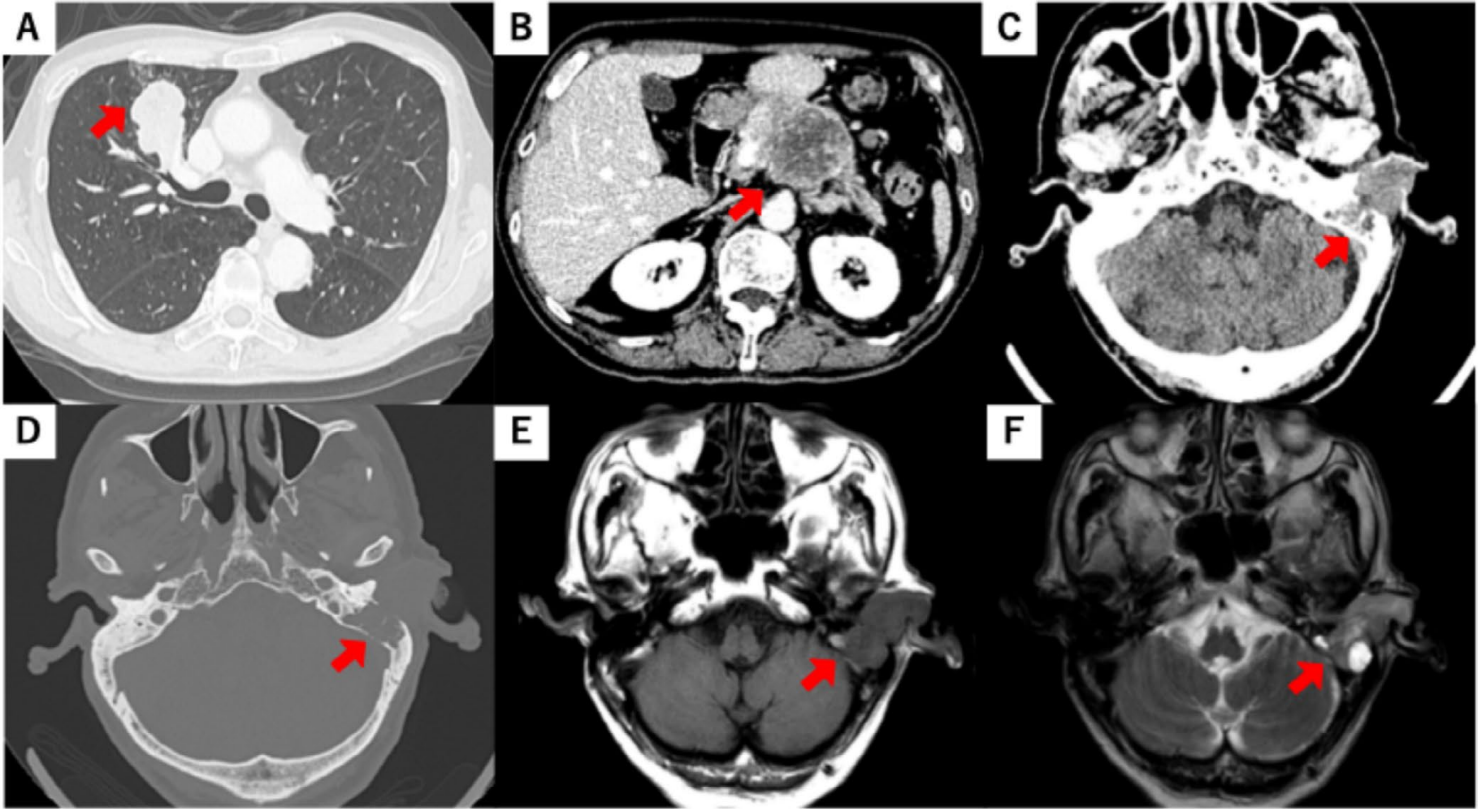
Computed tomography findings. (A) The mass in right upper lobe of lung. (B) The mass in the pancreatic body. (C) The mass in the left external auditory canal. (D) Destruction of left mastoid. Magnetic resonance imaging findings. (E) T1‐weighted imaging. (F) T2‐weighted imaging.

**FIGURE 3:**
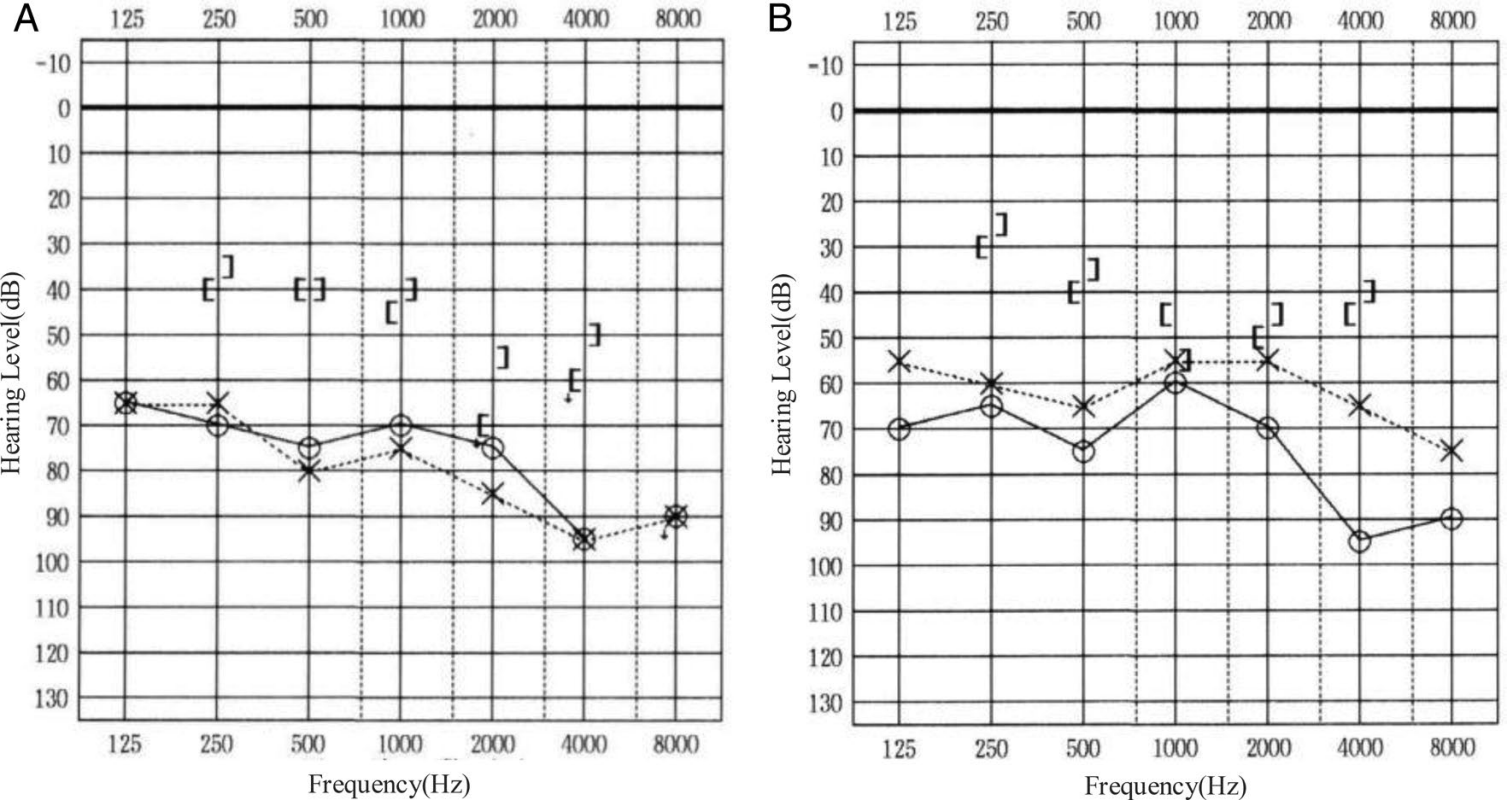
Audiometric results. (A) One month after chemotherapy. (B) Eight months after chemotherapy.

A 77‐year‐old man was presented with left‐sided hearing loss, otalgia, and a red mass in the left EAC that persisted for several weeks (Figure 1). His medical history included hypertension, hyperlipidemia, and right‐sided hearing loss due to chronic otitis media. He was a heavy smoker and had but no medical history of malignancy. Physical examination revealed a red, soft mass measuring approximately 20 mm occupying the left EAC. Computed tomography (CT) revealed masses in the left EAC and middle ear, as well as in the right upper lung lobe and pancreatic body (Figure 2). The mass in the EAC filled the entire canal and extended into the mastoid, as observed on both CT and magnetic resonance imaging (MRI). Laboratory tests showed normal Span‐1 and DUPAN2 levels, whereas ProGRP and NSE levels were elevated to 698 pg/mL and 31.1 ng/mL, respectively. Pure‐tone audiometry (PTA) revealed bilateral mixed hearing loss, with an average threshold of 72.5 dB in the right ear and 78.8 dB in the left ear at 500, 1000, 2000, and 4000 Hz (Figure 3).

**FIGURE 4:**
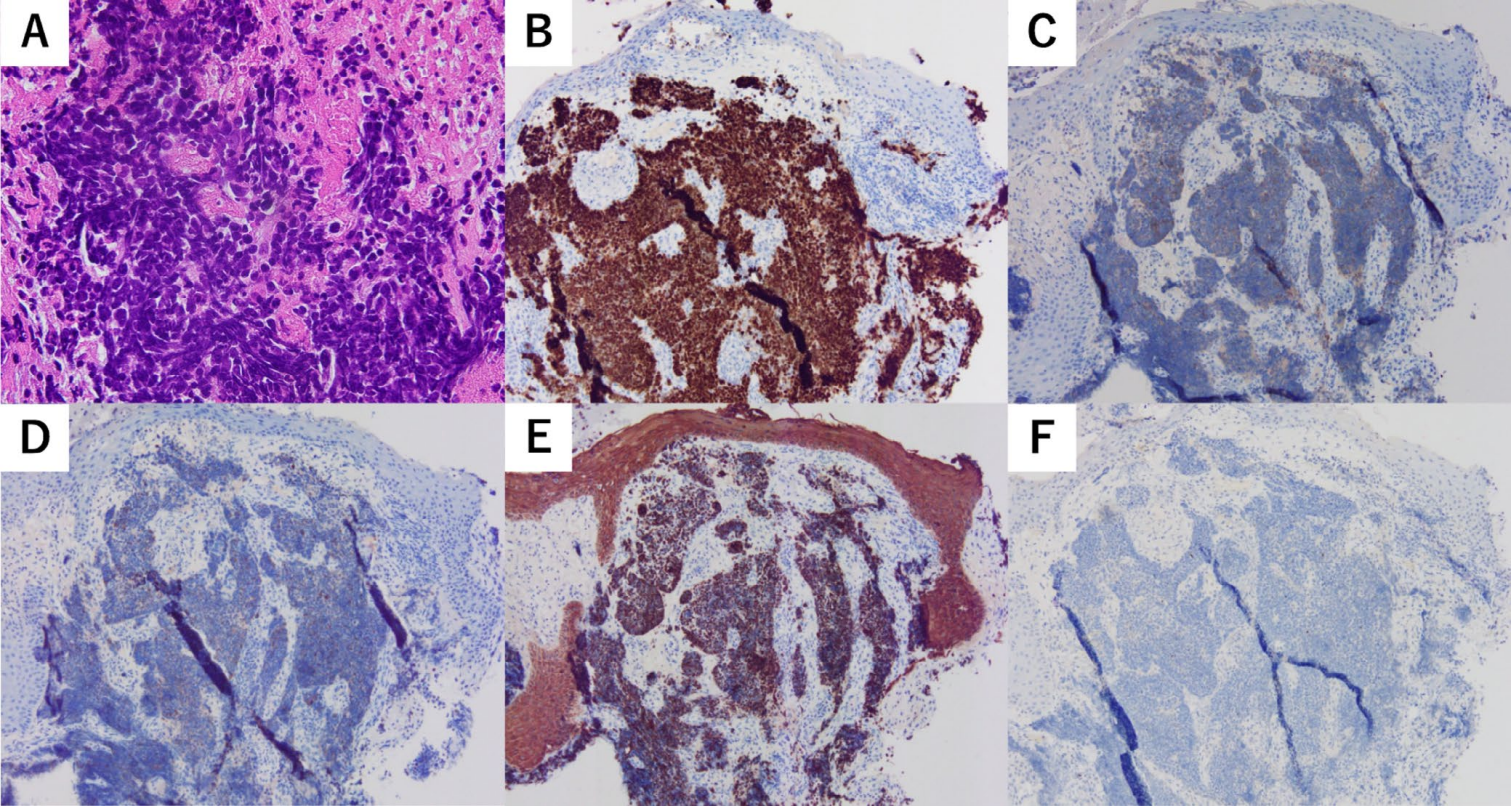
Histological findings of the left external auditory canal lesions. (A) Hematoxylin and eosin staining demonstrates small round cell tumors with high nuclear to cytoplasmic ratio. The tumor cells are (B) thyroid transcription factor‐1‐positive; (C) synaptophysin‐positive; (D) chromogranin A‐positive; (E) cytokeratin AE1/3‐positive; (F) CK20‐negative.

Biopsies were obtained from the EAC, lung, and pancreatic lesions to differentiate between primary and metastatic EAC carcinoma. Hematoxylin and eosin staining of all lesions revealed small round cell tumors with a high nuclear‐to‐cytoplasmic ratio (Figure 4). Immunohistochemical staining demonstrated positivity for synaptophysin, chromogranin A, and cytokeratin AE1/3, whereas CK20 was negative in all lesions. The biopsy specimens confirmed small cell lung cancer (SCLC), and thyroid transcription factor‐1 (TTF‐1) positivity in the EAC and pancreatic lesions suggested a primary tumor in the lung. Chemotherapy with carboplatin, etoposide, and zoledronic acid for bone metastases reduced the size of metastatic lesions, including those in the EAC and middle ear. Hearing impairment and otalgia also improved, with PTA showing an improvement in left‐sided mixed hearing loss, reducing the average threshold to 57.5 dB. The patient died 15 months later, and autopsy findings revealed that EAC lesions had resolved grossly.

## 3 Discussion

We encountered an unusual case of SCLC with EAC metastasis, where chemotherapy effectively improved hearing impairment and otalgia. To the best of our knowledge, only three prior reports have documented lung cancer metastasis to the EAC [2, 3, 4].

Three major mechanisms contribute to temporal bone metastasis, including involvement of the EAC: hematogenous spread, direct tumor extension, and diffuse leptomeningeal carcinomatosis [5, 6, 7]. In this case, hematogenous dissemination likely caused the metastasis, as no evidence of direct extension or meningitis was identified. SCLC frequently spreads via the bloodstream, and the absence of lymph node metastasis, including the mediastinum, supports this mechanism in this case. Previous studies indicated that metastases most commonly affect the petrous apex (82.9%) and mastoid (27.6%). Sluggish blood flow in the sinusoidal capillaries of the bone marrow favors the deposition of tumor cell deposition in the temporal bone [7, 8, 9]. CT imaging revealed mastoid destruction and tumor invasion into the EAC.

Patients with EAC metastases often present with hearing loss and ear bleeding. Histological classifications of lung cancer involving the EAC frequently include poorly differentiated adenocarcinoma or SCLC. The prognosis remains poor, with previous cases reporting survival of approximately 6 months. Conversely, this patient survived for 15 months. The favorable response to initial chemotherapy suggests that response to first‐line treatment plays a crucial role in overall survival. SCLC typically exhibits high sensitivity to chemotherapy, producing significant tumor reduction [10, 11, 12]. Initially, the patient experienced severe hearing loss and regained the ability to understand conversations as the tumor shrank. Otalgia also resolved, allowing the discontinuation of opioid therapy. Although multimodal therapy is often necessary for EAC malignancies, chemotherapy alone may effectively improve symptom and quality of life when tumors exhibit high sensitivity to anticancer drugs [13, 14, 15]. The introduction of immune checkpoint inhibitors (ICI) in combination with chemotherapy may further enhance therapeutic outcomes for EAC metastases.

Although temporal bone metastases, including those affecting the EAC, remain rare, these cases may be underdiagnosed due to symptom overlap with chronic otitis media and mastoiditis [16, 17]. Advances in imaging technology and novel anticancer therapies are expected to improve the detection and treatment of EAC metastases.

In conclusion, metastatic lung cancer should be considered in patients with unexplained otalgia or hearing loss. Routine otoscopic examinations may aid in early diagnosis for individuals with advanced lung cancer patients presenting with atypical symptoms. A strong response to initial chemotherapy plays a critical role in prolonging overall survival, and chemotherapy targeting highly sensitive EAC metastases may improve both symptoms and overall prognosis.

## Author Contributions

**Toshiyuki Ito:** conceptualization, data curation, investigation, project administration, resources, visualization, writing – original draft preparation, writing – review and editing. **Masamichi Yoshida:** methodology. **Hiroto Miki:** data curation, investigation. **Hiroki Goto:** data curation, investigation. **Shuuji Kodama:** data curation, investigation. **Atsushi Fujiwara:** investigation, resources. **Hajime Fujimoto:** conceptualization, investigation, resources. **Tetsu Kobayashi:** investigation, resources, supervision.

## Disclosure

The authors have nothing to report.

## Ethics Statement

Although not required by the ethics committee of our research institution because of the case report, written informed consent was obtained from the patient.

## Consent

Written informed consent was obtained from the patient for publication of this case report and accompanying images.

## Conflicts of Interest

The authors declare no conflicts of interest.

## Data Availability

The datasets generated during and analyzed during the current case report are available from the corresponding author on reasonable request.

## References

[1] H. Sung , J. Ferlay , R. Siegel , et al., “Global Cancer Statistics 2020: GLOBOCAN Estimates of Incidence and Mortality Worldwide for 36 Cancers in 185 Countries,” CA: A Cancer Journal for Clinicians 71 (2021): 209–249. 33538338 10.3322/caac.21660

[2] S. Ayadi , O. Walha , R. Kharrat , et al., “External Auditory Canal Metastasis Revealing Bronchogenic Carcinoma: A Case Report and Literature Review,” SAGE Open Medical Case Reports 12 (2024): 1–6. 10.1177/2050313X241258155 PMC11143865 38828381

[3] I. Vasileiadis , S. Kapetanakis , D. Vasileiadis , A. Petousis , and T. Karatzas , “External Auditory Canal Mass as the First Manifestation of a Bronchogenic Carcinoma: Report of a Rare Case,” Annals of Otology, Rhinology, and Laryngology 122 (2013): 378–381. 23837390 10.1177/000348941312200606

[4] M. Hain , E. Feldberg , and D. Halperin , “Case Report: Metastatic Small‐Cell Lung Carcinoma of the External Auditory Canal,” Ear, Nose, & Throat Journal 91 (2012): 476–478. 10.1177/014556131209101107 23288792

[5] H. F. Schuknecht , A. F. Allam , and Y. Murakami , “Pathology of Secondary Malignant Tumors of the Temporal Bone,” Annals of Otology, Rhinology, and Laryngology 77 (1967): 5–22. 10.1177/000348946807700101 5645142

[6] T. Hoshino , F. Hiraide , and Y. Nomura , “Metastatic Tumor of the Inner Ear: A Histopathological Report,” Journal of Laryngology and Otology 86 (1972): 697–707. 5044788 10.1017/s0022215100075757

[7] N. T. Berlinger , S. Koutroupas , G. Adams , and R. Maisel , “Patterns of Involvement of the Temporal Bone in Metastatic and Systemic Malignancy,” Laryngoscope 90 (1980): 619–627. 7359981 10.1288/00005537-198004000-00008

[8] T. I. Gloria‐Cruz , P. A. Schachern , M. M. Paparella , G. L. Adams , and S. E. Fulton , “Metastases to the Temporal Bones From Primary Nonsystemic Malignant Neoplasms,” Archives of Otorhinolaryngology‐Head & Neck Surgery 126 (2000): 209–214. 10.1001/archotol.126.2.209 10680873

[9] B. Proctor and J. R. Lindsay , “Tumors Involving the Petrous Pyramid of the Temporal Bone,” Archives of Otolaryngology 46 (1947): 180–194. 20260370 10.1001/archotol.1947.00690020189005

[10] K. Noda , Y. Nishiwaki , M. Kawaraha , et al., “Irinotecan Plus Cisplatin Compared With Etoposide Plus Cisplatin for Extensive Small‐Cell Lung Cancer,” New England Journal of Medicine 346, no. 2 (2002): 85–91, 10.1056/NEJMoa003034. 11784874

[11] H. Okamoto , K. Watanabe , H. Kunikane , et al., “Randomised Phase III Trial of Carboplatin Plus Etoposide vs Split Doses of Cisplatin Plus Etoposide in Elderly or Poor‐Risk Patients With Extensive Disease Small‐Cell Lung Cancer: JCOG 9702,” British Journal of Cancer 92 (2007): 162–169. 10.1038/sj.bjc.6603810 PMC2360311 17579629

[12] M. Satouchi , Y. Kotani , T. Shibata , et al., “Phase III Study Comparing Amrubicin Plus Cisplatin With Irinotecan Plus Cisplatin in the Treatment of Extensive‐Disease Small‐Cell Lung Cancer: JCOG 0509,” Journal of Clinical Oncology 32 (2014): 1262–1268. 24638015 10.1200/JCO.2013.53.5153

[13] R. Yasumatsu , K. Okura , Y. Sakiyama , et al., “Metastatic Hepatocellular Carcinoma of the External Auditory Canal,” World Journal of Gastroenterology 13, no. 47 (2007): 6436–6438, 10.3748/wjg.v13.i47.6436. 18081236 PMC4205466

[14] L. G. T. Morris , S. Mehra , J. P. Shah , M. H. Bilsky , S. H. Selesnick , and D. H. Kraus , “Predictors of Survival and Recurrence After Temporal Bone Resection for Cancer,” Head & Neck 34 (2012): 1231–1239. 21953902 10.1002/hed.21883 PMC4126564

[15] K. Ogawa , K. Nakamura , K. Hatano , et al., “Treatment and Prognosis of Squamous Cell Carcinoma of the External Auditory Canal and Middle Ear: A Multi‐Institutional Retrospective Review of 87 Patients,” International Journal of Radiation Oncology, Biology, Physics 68 (2007): 1326–1334. 17446002 10.1016/j.ijrobp.2007.01.052

[16] E. G. Nelson and R. Hinojosa , “Histopathology of Metastatic Temporal Bone Tumors,” Archives of Otolaryngology – Head & Neck Surgery 117 (1991): 189–193. 1991061 10.1001/archotol.1991.01870140077010

[17] A. L. Morton , S. A. Butler , A. Khan , A. Johnson , and P. Middleton , “Temporal Bone Metastases—Pathophysiology and Imaging,” Otolaryngology and Head and Neck Surgery 97 (1987): 583–587. 10.1177/019459988709700612 2829099

